# Supplementary material for: Chemical Composition and Antimicrobial Activity of New Honey Varietals
Source: Int J Environ Res Public Health. 2023 Jan 30;20(3):2458. doi: 10.3390/ijerph20032458 (PMC9915547; doi:10.3390/ijerph20032458)
Supplement: Supplementary file 1 [file ijerph-20-02458-s001.zip › ijerph-2114430-supplementary.pdf]

Supplementary materials

Table S1. Honeys' abbreviation. Type of honey determined on the dominant pollen or most common pollen.

No.	Honey sample	The acronym of the name of honey in article	The most common plant pollen/pollens in a given honey	The flowering period of the plant	The place of origin of the honey
1	Plum	P	*Prunus spinosa* L.	April-May	Lublin city, Urban Artistic Apiary, 51°14′53″N 22°34′13″E
2	Multifloral-AP	MAP	*Acer negundo* L., *Prunus spinosa* L.	April-May	Lublin city, Urban Artistic Apiary, 51°14′53″N 22°34′13″E
3	Multifloral-P	MP	*Prunus spinosa* L.	April-May	suburban areas, Nowy Gaj 51°15′43″N 22°13′46″E
4	Multifloral-Sa	MSa	*Salix* sp.	April-June	suburban areas, Czerniejów 51°07′16,5″N 22°35′59,5″E
5	Willow	Sa	*Salix* sp.	April-June	ecologically clean areas of Podkarpackie voivodeship 49°47′40″N, 21°56′54″E
6	Multifloral-Br	MBr	*Brassica napus* L.	May-June	Lublin city, Urban Artistic Apiary, 51°14′53″N 22°34′13″E
7	Rapeseed	Br	*Brassica napus* L.	May-June	suburban areas, Nowy Gaj 51°15′43″N 22°13′46″E
8	Lime	Tc	*Tilia cordata* Mill.	June-July	Lublin city, Urban Artistic Apiary, 51°14′53″N 22°34′13″E
9	Phacelia	Ph	*Phacelia tanacetifolia* Benth.	June-October	ecologically clean areas of Podkarpackie voivodeship 49°47′40″N, 21°56′54″E
10	Honeydew	So	*Solidago virgaurea* L.	August-October	ecologically clean areas of Podkarpackie voivodeship 49°47′40″N, 21°56′54″E
11	Sunflower	He	*Helianthus* sp.	August-November	ecologically clean areas of Lubelskie voivodeship (around the Polesie National Park), 51°27′19″N 23°10′24″E

Table S2. Mellisopalynological analysis of different types of honey

Nectar producing plant taxons	HONEY TYPE																					
	P		MAP		MP		MSa		Sa		MBr		Br		Tc		Ph		So		He	
	Pollen grains																					
	No.	%	No.	%	No.	%	No.	%	No.	%	No.	%	No.	%	No.	%	No.	%	No.	%	No.	%
Acer sp.	1	0.47	200	37.38	6	3.16			1	0.21												
Achillea millefolium															1	0.21	1	0.26				
Aesculus hippocastanum	28	13.02	35	6.54			12	10.34	1	0.21	16	15.53			55	11.39	5	1.31				
Anthriscus sp.											1	0.97			5	1.04			5	10.87	1	0.24
Arctium sp.																					1	0.24
Aster type											4	3.88					1	0.26				
Brassicaceae type	13	6.05			30	15.79					34	33.01	260	81.76	41	8.49	22	5.77			60	14.67
Centaurea cyanus			25	4.67													5	1.31				
Cirsium sp.															2	0.41						
Convolvulus arvensis	1	0.47																				
Echium vulgare											1	0.97										
Fagopyrum esculentum	3	1.40																			1	0.24
Frangula alnus									2	0.41	1	0.97									3	0.73
Helianthus type	26	12.09									7	6.80			6	1.24			1	2.17	300	73.35
Impateins sp.																			5	10.87		
Lilium sp.					5	2.63	1	0.86														
Lotus corniculatus									3	0.62												
Mallus type	7	3.26			15	7.89			1	0.21					8	1.66						
Melilotus sp.																	50	13.12				
Phacelia thanacetifolia					5	2.63	18	15.52			2	1.94			3	0.62	250	65.62			2	0.49
Poligonum bistorta			5	0.93	4	2.11					16	15.53			130	26.92						
Prunus type	101	46.98	200	37.38	56	29.47			130	26.86			20	6.29	23	4.76	3	0.79			7	1.71
Robinia pseudoacacia											1	0.97			7	1.45						

Rubus sp.					15	7.89	10	8.62			1	0.97	20	6.29	25	5.18	4	1.05	15	32.61	6	1.47
Salix sp.	31	14.42			29	15.26	25	21.55	340	70.25			18	5.66	2	0.41					13	3.18
Sedum sp.															2	0.41						
Solidago type	1	0.47					20	17.24							23	4.76	40	10.50	20	43.48	6	1.47
Trifolim repens	2	0.93																				
Trifolium pratense							10	8.62	1	0.21	4	3.88			10	2.07						
Taraxacum officinale	1	0.47	20	3.74																	4	0.98
Tilia sp.			50	9.35	20	10.53	20	17.24	4	0.83	15	14.56			140	28.99						
Viola type									1	0.21												
Others																					5	1.22
SUM/AMOUNT	215	95.98	535	95.54	190	59.38	116	43.61	484	98.57	103	89.74	318	93.53	483	76.86	381	98.45	46	38.2	409	97.15
**Not nectarative plant taxons**																						
Artemisia sp.	2	22.22					60	40			4	28.57			110	74.32						
Bellis perennis	1	11.11			5	2.63																
Betula pendula	1	11.11													1	0.68						
Chenopodiaceae type	2	22.22									2	14.29			2	1.35					6	50
Filipendula sp.			20	80%	130	100	90	60					20	90.91					50	66.67		
Fragaria sp.															1	0.68						
Pinus sp.			5	20					2	28.57			2	9.09			1	16.67				
Plantago sp.											4	28.57			17	11.49						
Poaceae type	2	22.22							5	71.43	4	28.57			12	8.11			25	33. 33		
Rumex sp.															5	3.38						
Quercus sp.	1	11.11																			6	50
Verbascum sp.																	5	83.33				
SUM/AMOUNT	9	4.02	25	4.46	130	40.63	150	56.39	7	1.43	14	10.26	22	6.47	148	23.14	6	1.55	75	66.96	12	2.85

Table S3. Color designations of honey after [1].

USDA color standard designation	Color range Pfund scale (mm)	Sample result range
Water White	≤ 8	0.0 – 0.094
Extra White	>8 and ≤ 17	0.0094 – 0.189
White	>17 and ≤ 34	0.189 – 0.378
Extra Light Amber	>34 and ≤ 50	0.378 – 0.595
Light Amber	>50 and ≤ 85	0.595 – 1.389
Amber	>85 and ≤ 114	1.389 – 3.008
Dark Amber	>114	> 3.008

Table S4. Colour of tested honey.

Honey type	Sample result (Absorbance) (average ± SD, N = 3)	Color
P	0.83 ± 0.01	Light Amber
MAP	2.94 ± 0.01	Amber
MP	1.36 ± 0.00	Light Amber
MSa	1.05 ± 0.01	Light Amber
Sa	0.42 ± 0.01	Extra Light Amber
MBr	0.74 ± 0.01	Light Amber
Br	1.76 ± 0.01	Amber
Tc	0.97 ± 0.01	Light Amber
Ph	1.85 ± 1.12	Amber
So	2.23 ± 0.01	Amber
He	0.36 ± 0.01	White

Antimicrobial activity of honey

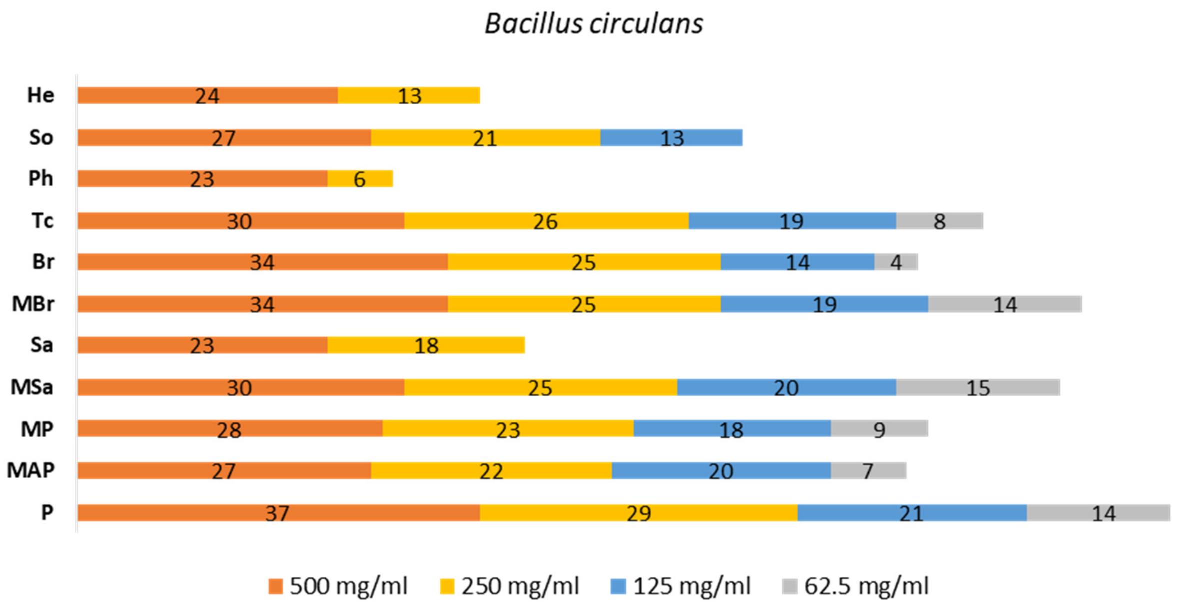

Figure S1. Antimicrobial activity of honey. Differentiation of inhibitory activity various concentrations of honeys.

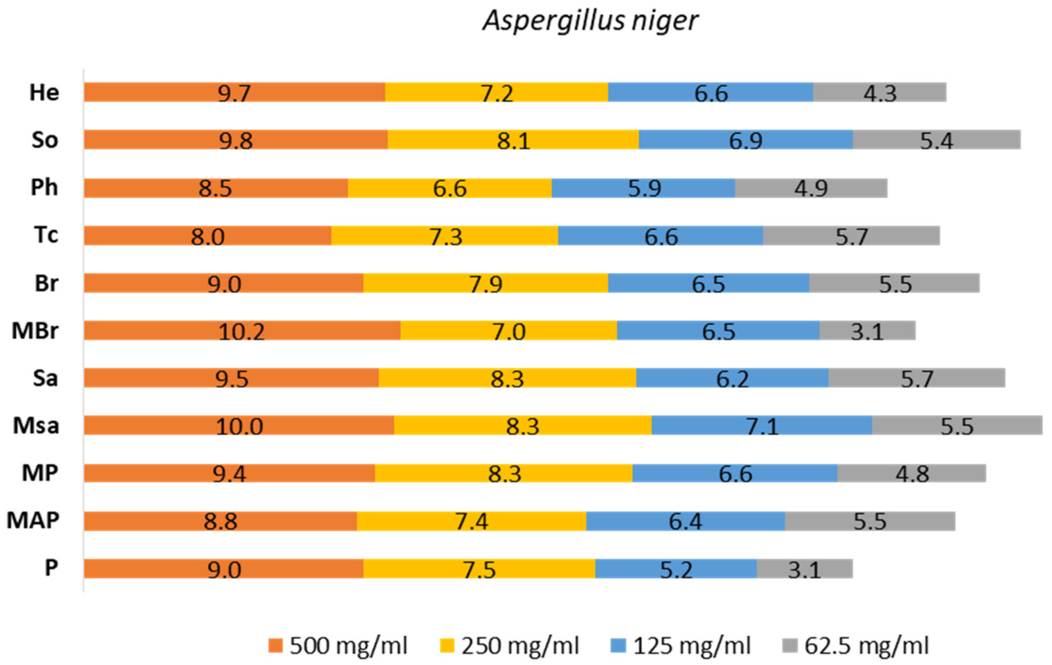

Figure S2. Antimicrobial activity of honey. Inhibitory activity of tested honey types relative to *A. niger*.

Checking the antimicrobial activity connected with catalase

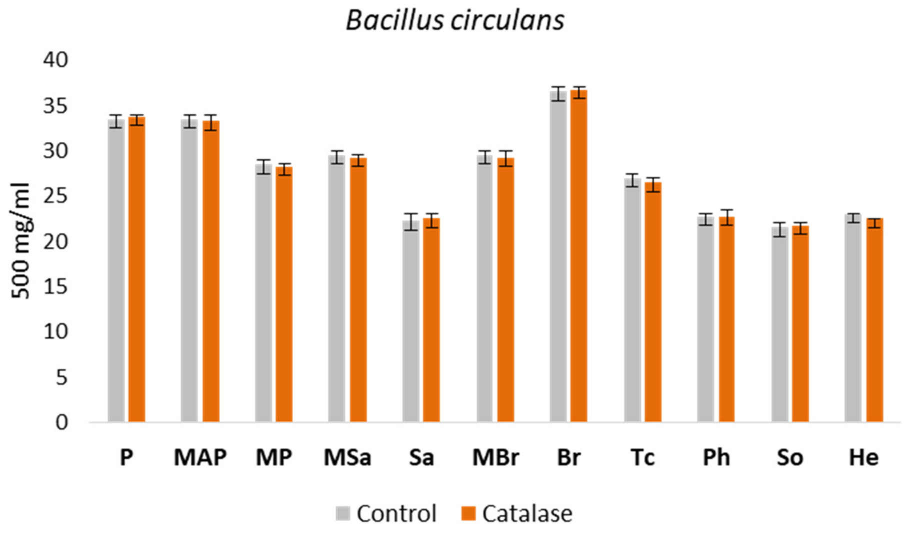

Figure S3. Catalase. Inhibitory activity of tested honey to *B. circulans*. The control sample only contains honey. The test sample contains honey with catalase at a final concentration of 0.2% (w/v). The error bars show standard deviation (SD).

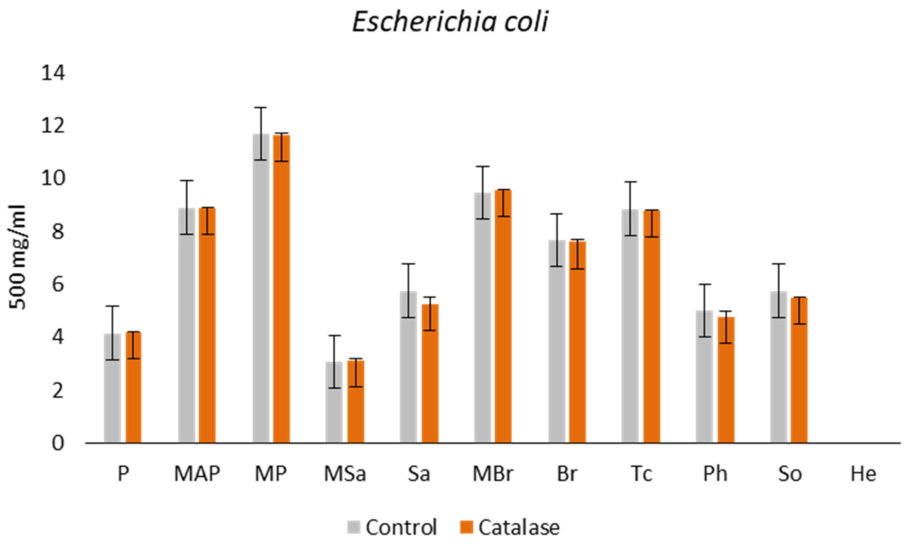

Figure S4. Catalase. Inhibitory activity of tested honey to *E. coli*. The control sample only contains honey. The test sample contains honey with catalase at a final concentration of 0.2% (w/v). The error bars show standard deviation (SD).

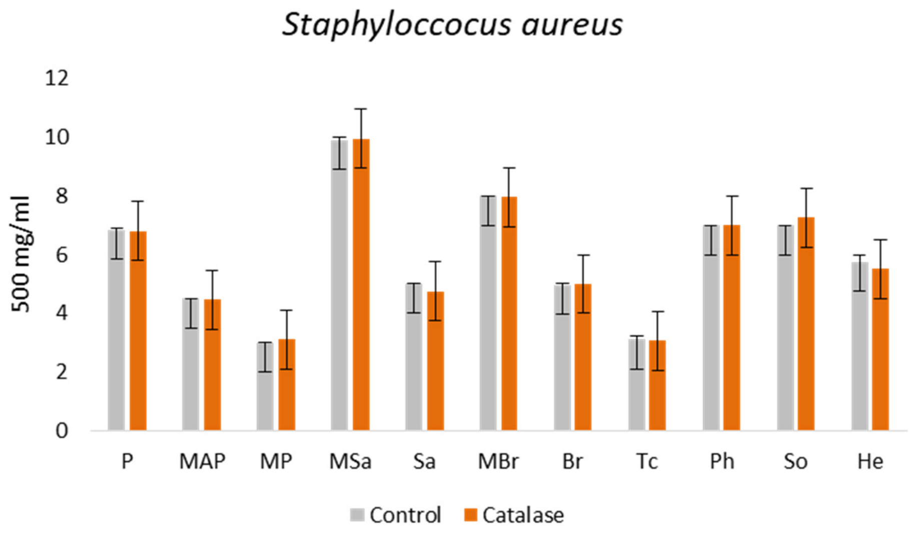

Figure S5. Catalase**.** Inhibitory activity of tested honey to *S. aureus*. The control sample only contains honey. The test sample contains honey with catalase at a final concentration of 0.2% (w/v). The error bars show standard deviation (SD).

Antimicrobial activity connected with hydrogen peroxide in honey samples

Agar plates (0.7%) with the LB medium (10 ml) (LB; Biocorp, Warszawa, Poland) containing appropriate bacterium (150 µl) in the amount of 1.5-4.2x10^6 were used to detect the antimicrobial activity connected with hydrogen peroxide in honey samples. Each well on the Petri plates was filled with 5 µl samples containing appropriate honey dilutions as a control and 5 µl samples containing appropriate honey dilutions with catalase (the enzyme degrading hydrogen peroxide) (Sigma-Aldrich, Saint Louis, MO, USA), as a test samples. Next, plates were incubated at 37°C for 24 hours and the diameters of bacterial growth inhibition zones were measured with digital caliper (Pro, Bielsko-Biała, Poland).

Positive control samples: Each well on the Petri plates was filled with 5 µl of freshly diluted 10%, 5%, 3%, 1,5% hydrogen peroxide (Chempur, H_2O_2 -34.01 g/mol 30% pure p.a. CAS: 7722-84-1) diluted in sterile water or honey (no. 11), next the agar plates were incubated for 24 hours at 37°C. The diameters of bacteria growth inhibition zones (Fig. S6.) were measured with digital caliper (Pro, Bielsko-Biała, Poland) and expressed in milimeters (Tab. S5). The experiment was repeated three times.

Escherichia coli

- 10% hydrogen peroxide (5 µl 10% hydrogen peroxide diluted in dd H20)
- 5% hydrogen peroxide (2.5 µl 10% hydrogen peroxide + 2.5 µl dd H_2O)
- 5% hydrogen peroxide (2.5 µl 10% hydrogen peroxide + 2.5 µl honey no. 11)
- 3% hydrogen peroxide (5 µl 3% hydrogen peroxide diluted in dd H20)
- 1.5% hydrogen peroxide (2.5 µl 3% hydrogen peroxide + 2.5 µl dd H_2O)
- 1.5% hydrogen peroxide (2.5 µl 3% hydrogen peroxide + 2.5 µl honey no. 11)

Staphylococcus aureus

- 10% hydrogen peroxide (5 µl 10% hydrogen peroxide diluted in dd H20)
- 5% hydrogen peroxide (2.5 µl 10% hydrogen peroxide + 2.5 µl dd H_2O)
- 5% hydrogen peroxide (2.5 µl 10% hydrogen peroxide + 2.5 µl honey no. 11)
- 3% hydrogen peroxide (5 µl 3% hydrogen peroxide diluted in dd H20)
- 1.5% hydrogen peroxide (2.5 µl 3% hydrogen peroxide + 2.5 µl dd H_2O)
- 1.5% hydrogen peroxide (2.5 µl 3% hydrogen peroxide + 2.5 µl honey no. 11)

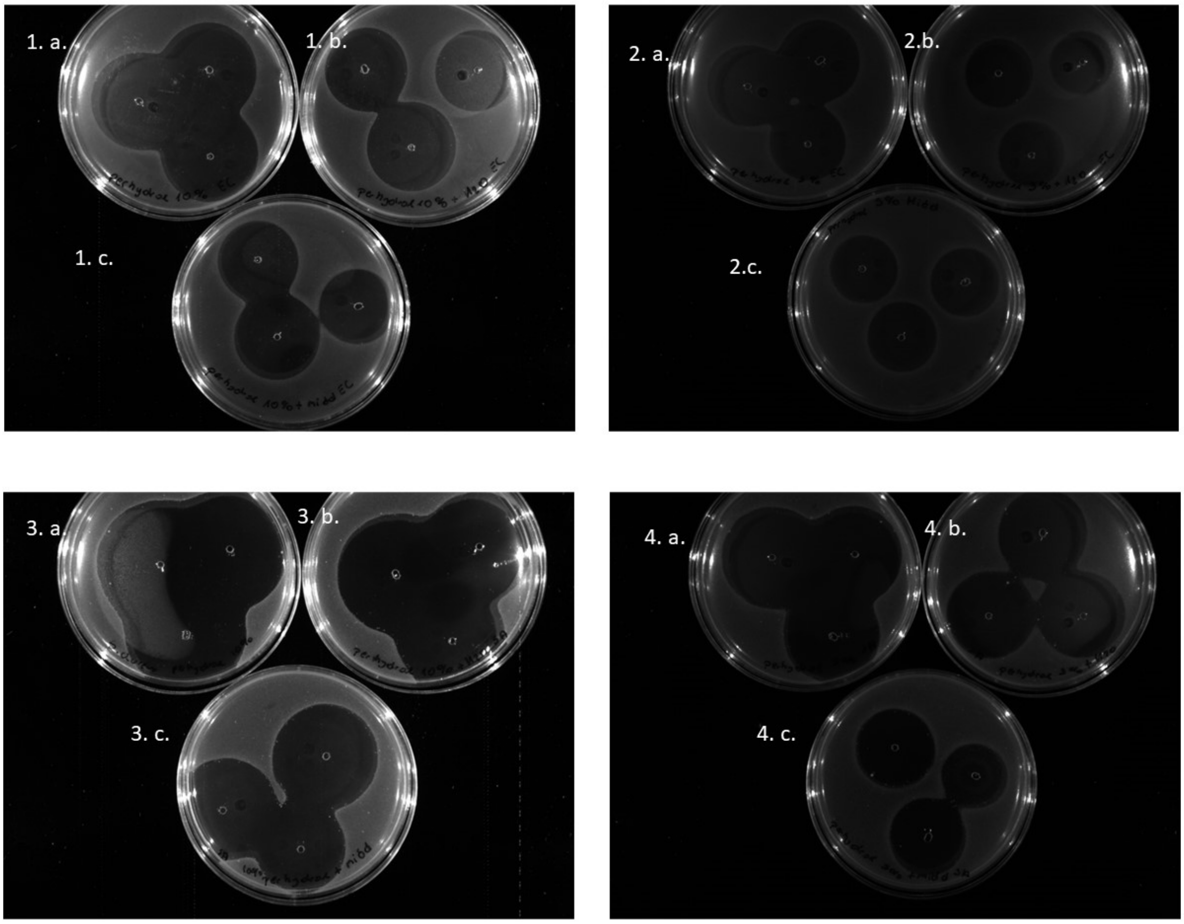

Figure. S6. Antimicrobial activity connected with hydrogen peroxide in honey samples. Positive control: bacteria growth inhibition zones.

Escherichia coli

1.a. – 10% hydrogen peroxide (5 µl 10% hydrogen peroxide diluted in dd H20)
1.b. – 5% hydrogen peroxide (2.5 µl 10% hydrogen peroxide + 2.5 µl dd H_2O)
1.c. – 5% hydrogen peroxide (2.5 µl 10% hydrogen peroxide + 2.5 µl honey no. 11)
2.a. – 3% hydrogen peroxide (5 µl 3% hydrogen peroxide diluted in dd H20)
2.b. – 1.5% hydrogen peroxide (2.5 µl 3% hydrogen peroxide + 2.5 µl dd H_2O)
2.c. – 1.5% hydrogen peroxide (2.5 µl 3% hydrogen peroxide + 2.5 µl honey no. 11)

Staphylococcus aureus

3.a. – 10% hydrogen peroxide (5 µl 10% hydrogen peroxide diluted in dd H20)

3.b. – 5% hydrogen peroxide (2.5 µl 10% hydrogen peroxide + 2.5 µl dd H_2O)
3.c. – 5% hydrogen peroxide (2.5 µl 10% hydrogen peroxide + 2.5 µl honey no. 11)
4.a. – 3% hydrogen peroxide (5 µl 3% hydrogen peroxide diluted in dd H20)
4.b. – 1.5% hydrogen peroxide (2.5 µl 3% hydrogen peroxide + 2.5 µl dd H_2O)
4.c. – 1.5% hydrogen peroxide (2.5 µl 3% hydrogen peroxide + 2.5 µl honey no. 11)

Table S5. Positive control: diameters of bacteria growth inhibition zones [mm].

Escherichia coli	Repetition 1	Repetition 2	Repetition 3	**Mean**
10% hydrogen peroxide (5 µl 10% hydrogen peroxide diluted in dd H20)	36.1	36.6	36.4	**36.37**
5% hydrogen peroxide (2.5 µl 10% hydrogen peroxide + 2.5 µl dd H_2O)	31.7	30.4	33	**31.70**
5% hydrogen peroxide (2.5 µl 10% hydrogen peroxide + 2.5 µl honey no. 11)	32	28.1	30.4	**30.17**
3% hydrogen peroxide (5 µl 3% hydrogen peroxide diluted in dd H20)	29.5	30.3	30.5	**30.10**
1,5% hydrogen peroxide (2.5 µl 3% hydrogen peroxide + 2.5 µl dd H_2O)	23	25.5	26	**24.83**
1.5% hydrogen peroxide (2.5 µl 3% hydrogen peroxide + 2.5 µl honey no. 11)	26.6	26.5	25.8	**26.30**
Staphylococcus aureus				
10% hydrogen peroxide (5 µl 10% hydrogen peroxide diluted in dd H20)	44.5	46	45.6	**45.37**
5% hydrogen peroxide (2.5 µl 10% hydrogen peroxide + 2.5 µl dd H_2O)	40	41.2	43.8	**41.67**
5% hydrogen peroxide (2.5 µl 10% hydrogen peroxide + 2.5 µl honey no. 11)	40	38.4	35.5	**37.97**
3% hydrogen peroxide (5 µl 3% hydrogen peroxide diluted in dd H20)	37	38.2	37.4	**37.53**
1.5% hydrogen peroxide (2.5 µl 3% hydrogen peroxide + 2.5 µl dd H_2O)	34.4	34.5	33.2	**34.03**
1.5% hydrogen peroxide (2.5 µl 3% hydrogen peroxide + 2.5 µl honey no. 11)	31	30	26	**29.00**

Lysozyme-like activity - Methods

1. *Preparation of honey samples*

The protein extraction of honey samples was carried out by the method of Baroni et al. [2]. Ten grams of rapeseed honey obtained in 2022 from the Lubelskie voivodeship was suspended in 10 mL of distilled water and proteins were extracted by overnight shaking at 4°C. Next, the mixture was centrifuged at 21,000g for 45 minutes at 4°C. The supernatant was dialyzed overnight in deionized water. The samples was frozen and lyophilized and stored at -20°C for further analysis.

2. *SDS-PAGE*

Poliacrylamide gel electrophoresis of proteins and peptides were performed by 13.8% Tris-glycine SDS-PAGE according to Laemmli [3]. The gels were stained with 0.1% solution of Coomassie Brilliant Blue R-250 for 45 minutes and then destained in 10% acetic acid.

3. *Protein content*

The protein concentration was determined using bovine serum albumin (BSA) as a standard according to Bradford [4].

4. *Determination of lysozyme-like activity of honey samples*

Lysozyme-like activity in the honey samples was checked using agarose plates containing freeze-dried *Micrococcus lysodeikticus* (Sigma) [5].

The activity was tested in the following steps of preparation of the honey samples:

- In the mixture after overnight shaking and centrifuged (sample 1)
- In the supernatant after dialysis (sample 2)
- In the lyophilized samples (sample 3)

Each well on the Petri plates was filled with 5 μl samples, next plates were incubated at 28°C for 24 hours. After this time, peptidoglycan digestion zones were measured. The lysozyme-like activity was defined as an equivalent of EWL activity (μg/ml) (Sigma, EC 3.2.1.17). Similarly, for control plates, wells were filled with egg white lysozyme (EWL).

Lysozyme-like activity level - Results

Lysozyme-like activity level was tested in all samples taken at various steps of honey preparation i.e. after centrifuged, dialysis and lyophilization (Tab. 5). Peptidoglycan digestion zone was shown in Figures S1. The level of lysozyme-like activity was calculated on the basis of standard curve prepared for known concentrations of lysozyme EWL (μg/ml) (Sigma-Aldrich, Saint Louis, MO, USA) (Fig.S7-S8).

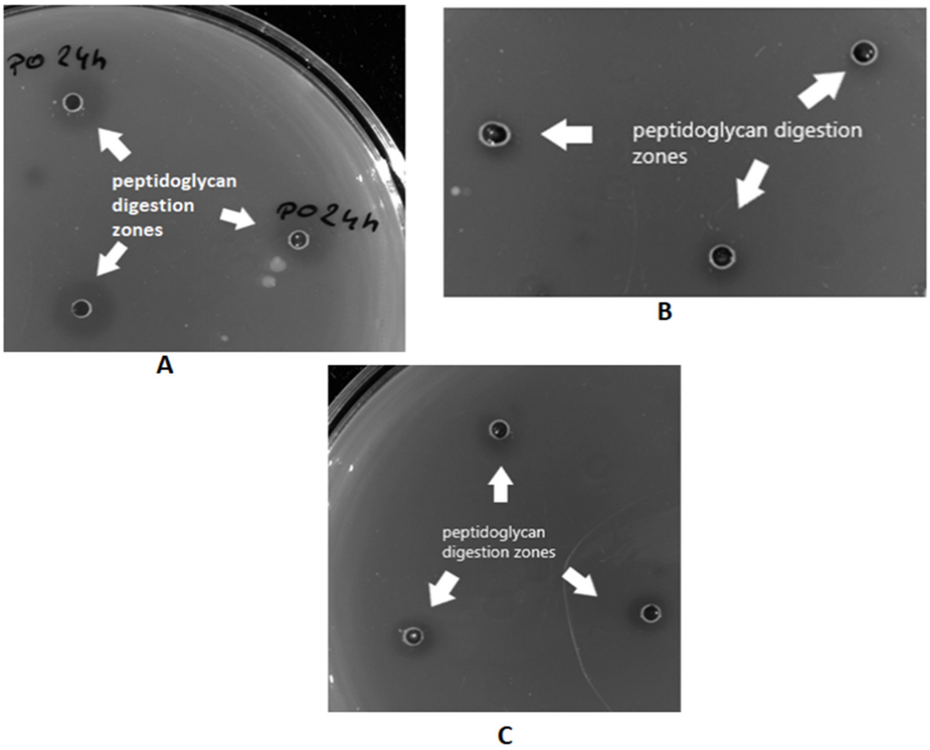

Figure S7. Lysozyme-like activity. Detection of lysozyme-like activity by the radial diffusion method in honey after centrifugation (A), dialysis (B) and lyophilization (C).

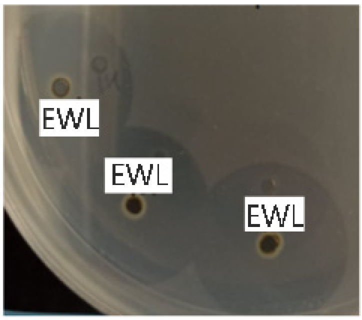

Figure S8. Lysozyme-like activity. Control plate containing egg white lysozyme (EWL).

SDS-PAGE analysis of honey proteins/peptides of the sample 3

Honey proteins were separated by electrophoresis and visualized on a polyacrylamide gel. A protein band of approximately 14 kDa, corresponding to the molecular mass of lysozyme, was distinguished [Fig S9].

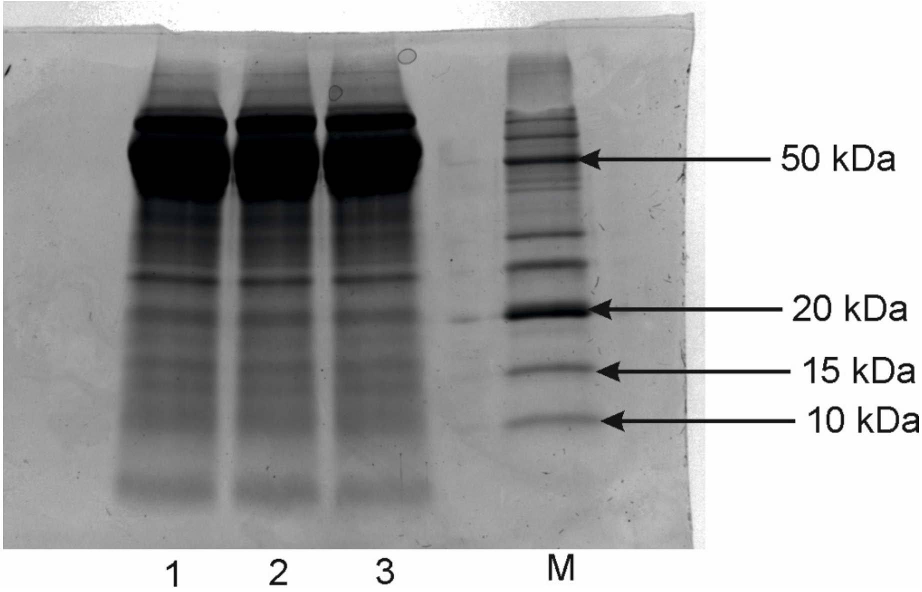

Figure S9. SDS-PAGE analysis of honey proteins/peptides (sample 3). The proteins were resolved by glycine SDS-PAGE and stained with Coomassie Brilliant Blue R-250. M- molecular mass marker, 1-2-3 – honey samples in triplicate, x –protein with a molecular mass of approximately 14 kDa.

Our preliminary results showed that there is activity against *M. lysodeikticus* at each step in the honey samples, which is defined as lysozyme-like activity. In order to find out whether there is lysozyme protein in honey, it is necessary to perform long-term experiments.

Sugar analysis in honey samples

A representative HPLC profile of honey no. 11 (Fig S10).

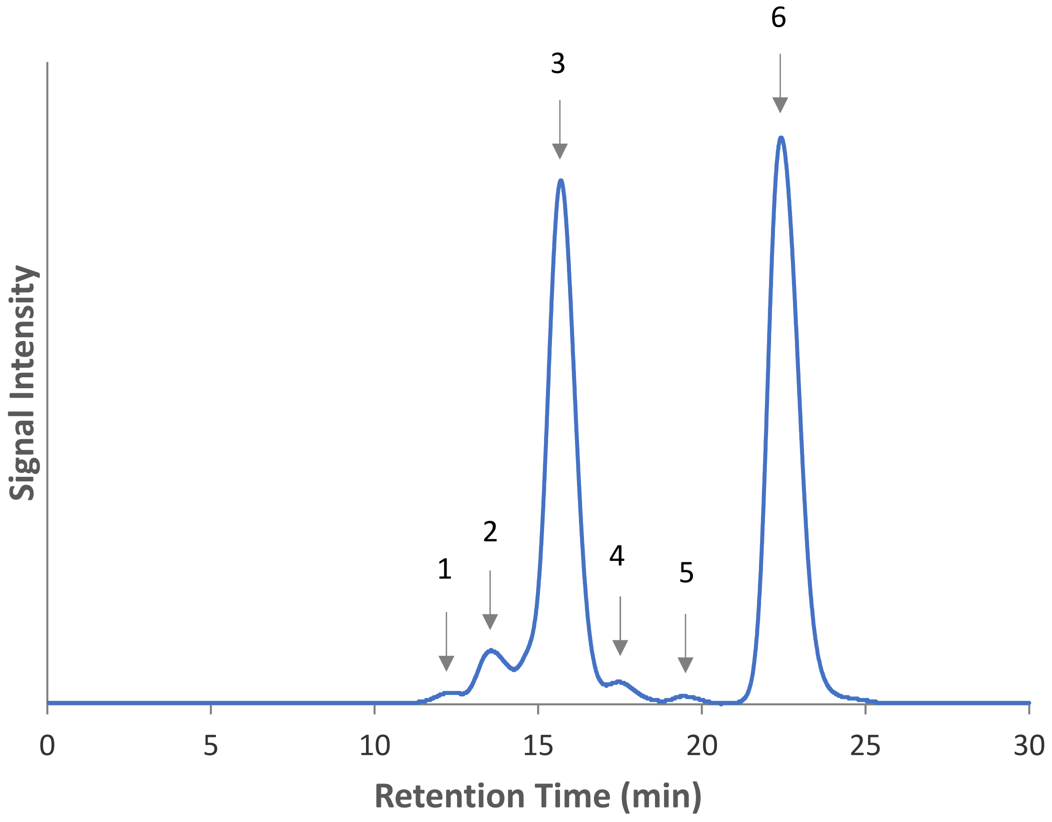

Figure S10. Sugar analysis in honey samples. Representative high performance liquid chromatography (HPLC) profile of honey no.11. Identified peaks are as follows: 1 - Erlose; 2 - Sucrose; 3 - Glucose; 4 - Rhamnose; 5 - Fucose; 6 - Fructose.

References:

1. Frasco D. Analysis of Honey Color and HMF Content using a Genesys UV-Visible Spectrophotometer, Thermo Fisher Scientific, Madison, WI, USA. 2018. Available from: https://assets.thermofisher.com/TFS-Assets/MSD/Application-Notes/honey-color-hmf-content-analysis-using-genesys-uv-visible-spectrophotometer-AN53025.pdf.
2. Baroni MV, Chiabrando GA, Costa C, Wunderlin DA. Assessment of the floral origin of honey by SDS-page immunoblot techniques. J Agric Food Chem. 2002. 13;50:1362-7. doi: 10.1021/jf011214i. PMID: 11879003.
3. Laemmli U.K. Cleavage of structural proteins during the assembly of the head of bacteriophage T4. Nature. 1970. 227, 680–685
4. Bradford MM. Rapid and sensitive method for quantification of microgram quantities of protein utilizing the principle dye binding. Anal Biochem. 1976, 72, 248-254. http://dx.doi. org/10.1016/0003-2697(76)90527-3.
5. Mohrig W, Messner B. Lyzozym als antibakterielles Agents im Bienenhonig und Bienengift. Acta Biol Med Germ. 1968;21: 85-90.
